# Online Detection of Fetal Acidemia during Labour by Testing Synchronization of EEG and Heart Rate: A Prospective Study in Fetal Sheep

**DOI:** 10.1371/journal.pone.0108119

**Published:** 2014-09-30

**Authors:** Xiaogang Wang, L. Daniel Durosier, Michael G. Ross, Bryan S. Richardson, Martin G. Frasch

**Affiliations:** 1 Department of Mathematics and Statistics, York University, Toronto, Ontario, Canada; 2 Department of Obstetrics and Gynecology and Department of Neurosciences, CHU Sainte-Justine Research Centre, Université de Montréal, Montreal, Quebec, Canada; 3 Department of Obstetrics & Gynecology, LA BioMed at Harbor-UCLA Med. Ctr., Torrance, California, United States of America; 4 Department of Obstetrics and Gynecology, Univ. Western Ontario, London, Ontario, Canada; 5 Animal Reproduction Research Centre (CRRA), Faculty of Veterinary Medicine, Université de Montréal, Montréal, QC, Canada; University of Tennessee Health Science Center, United States of America

## Abstract

Severe fetal acidemia during labour can result in life-lasting neurological deficits, but the timely detection of this condition is often not possible. This is because the positive predictive value (PPV) of fetal heart rate (FHR) monitoring, the mainstay of fetal health surveillance during labour, to detect concerning fetal acidemia is around 50%. In fetal sheep model of human labour, we reported that severe fetal acidemia (pH<7.00) during repetitive umbilical cord occlusions (UCOs) is preceded ∼60 minutes by the synchronization of electroencephalogram (EEG) and FHR. However, EEG and FHR are cyclic and noisy, and although the synchronization might be visually evident, it is challenging to detect automatically, a necessary condition for bedside utility. Here we present and validate a novel non-parametric statistical method to detect fetal acidemia during labour by using EEG and FHR. The underlying algorithm handles non-stationary and noisy data by recording number of abnormal episodes in both EEG and FHR. A logistic regression is then deployed to test whether these episodes are significantly related to each other. We then apply the method in a prospective study of human labour using fetal sheep model (n = 20). Our results render a PPV of 68% for detecting impending severe fetal acidemia ∼60 min prior to pH drop to less than 7.00 with 100% negative predictive value. We conclude that this method has a great potential to improve PPV for detection of fetal acidemia when it is implemented at the bedside. We outline directions for further refinement of the algorithm that will be achieved by analyzing larger data sets acquired in prospective human pilot studies.

## Introduction

Human clinical studies with umbilical cord blood gas and pH assessment at birth indicate an increasing risk for neonatal adverse outcome with severe acidemia (arterial pH values less than 7.00) and in turn for longer-term sequellae including cerebral palsy [1]–[3]. Additionally, growth restricted infants with chronic hypoxemia due to placental dysfunction are at a greater risk for concerning metabolic acidosis at birth and thereby subsequent adverse outcome due to superimposed intrapartum hypoxic insult [4] [5] [6]. In low risk labouring patients at term, ∼20% had histologic chorioamnionitis in the absence of clinical chorioamnionitis and showed 5–10 fold increases in umbilical cord blood cytokines [7] [8]. These findings indicate that a low grade inflammation occurs in a significant proportion of patients labouring at term. Today, electronic fetal heart rate (FHR) monitoring is the main stay for the assessment of fetal health during labour when there is increased risk for a compromise in fetal oxygenation due to restriction in uterine and/or umbilical blood flow with or without underlying chronic hypoxia or inflammation [9] [10]. This is based upon the well-known change in FHR with acute hypoxic insult, *i.e.*, FHR decelerations, and the absence of these is highly predictive for normal fetal blood gas/pH at birth [9], [10]. However, FHR monitoring as used clinically has a low positive predictive value (PPV) for concerning metabolic acidosis at birth. There has been considerable study over the past decade into complimentary monitoring techniques including computerized FHR data acquisition and pattern scoring, fetal pulse oximetry and ECG waveform analysis with additional monitored information for discerning fetal health [9] [10]. However, to date, none of these have become standard of care despite the fact that intrapartum electronic FHR monitoring is ever increasing and now used in more than 90% of the hospitals providing birthing care in Canada [9] [10].

We recently studied patterns of electrocorticogram (ECOG) recorded from supradural electrodes and FHR in the near term ovine fetus and the response to induced hypoxemia in a well-established model of human pregnancy. The fetus was subjected to repetitive umbilical cord occlusions (UCOs) as might be seen in human labour, to delineate the time-course for ECOG change with worsening acidemia and whether or not this has clinical utility [11]. Animals underwent a series of mild, moderate and severe UCOs with each series lasting 1 h or until fetal arterial pH<7.00, and with ECOG activity continuously monitored using power spectral analysis. Repetitive UCOs resulted in worsening acidemia, which became severe with fetal arterial pH decreasing from 7.36 to 6.90. There were consistent changes in ECOG activity with amplitude suppression and frequency increase during the UCOs when FHR decelerations began to be associated with pathological decreases in fetal arterial blood pressure. These changes in ECOG occurred on average 50 minutes prior to attaining a severe degree of acidemia with pH<7.00. As such, fetal ECOG activity is predictably altered with repetitive UCOs leading to worsening acidemia, and therefore may prove useful for improving the positive predictive value of FHR monitoring for concerning metabolic acidosis at birth.

Consequently, we have been studying the potential of monitoring the fetal electroencephalogram (EEG) recorded from scalp electrodes during labour in addition to the standard FHR monitoring to improve detection of fetal acidemia. It is important to determine whether fetal EEG monitoring can be added in a meaningful and efficient manner, *i.e.*, adding clear prognostic information at the bedside without causing confusion, and with affordable and “user friendly” technology. First, we have shown that fetal EEG can be recorded from the fetal scalp reliably [12]. Second, we demonstrated that a degree of synchronization occurs between the EEG signal and FHR with repetitive UCOs leading to worsening acidemia and in advance of severe acidemia [13]. For bedside implementation, a reliable, observer-independent and real-time or online detection mechanism of this EEG-FHR synchronization would be desirable. However, the intrinsic variations and noise embedded in the EEG and FHR signals present a significant challenge for traditional statistical analysis. For example, an acidotic fetus during labour may present with some manifestation of abnormal EEG and FHR patterns. This will break the assumption of time series stationarity for many statistical methods. Measurement errors and confounding factors make the analysis even more challenging, since what we observe might not truly reflect the actual EEG and/or FHR signals.

In this article, we present a novel statistical method to detect the synchronization that occurs between the EEG and FHR signals during repetitive UCOs leading to worsening acidemia under variable baseline conditions, *i.e.*, normoxia, chronic hypoxia and low-grade inflammation as might be seen clinically during labour with fetal growth restriction and placental chorioamnionitis [14] [15] [16] [17].

## Experiments and Data Acquisition

### 1 Surgical preparation

Twenty near-term ovine fetuses (123±2 days gestational age (GA), term = 145 days) of mixed breed were surgically instrumented. The anesthetic and surgical procedures and postoperative care of the animals have been previously described [11], [18]–[20]. Briefly, polyvinyl catheters were placed in the right and left brachiocephalic arteries, the cephalic vein, and the amniotic cavity. Stainless steel electrodes were sewn onto the fetal chest to monitor the electrocardiogram (ECG). A polyvinyl catheter was also placed in the maternal femoral vein. A modified FHR electrode with a double spiral was placed on fetal head to acquire EEG, as might be done during human labour. An inflatable silicon rubber cuff (In Vivo Metric, Healdsburg, CA) for UCO induction was placed around the proximal portion of the umbilical cord and secured to the abdominal skin. Once the fetus was returned to the uterus, a catheter was placed in the amniotic fluid cavity. Antibiotics were administered intravenously to the mother (0.2 g trimethoprim and 1.2 g sulfadoxine, Schering Canada Inc., Pointe-Claire, Canada) and the fetus and into the amniotic cavity (1 million IU penicillin G sodium, Pharmaceutical Partners of Canada, Richmond Hill, Canada). Amniotic fluid lost during surgery was replaced with warm saline. The uterus and abdominal wall incisions were sutured in layers and the catheters exteriorized through the maternal flank and secured to the back of the ewe in a plastic pouch.

Postoperatively, animals were allowed four days to recover prior to experimentation and daily antibiotic administration was continued intravenously to the mother (0.2 g trimethoprim and 1.2 g sulfadoxine), into the fetal vein and the amniotic cavity (1 million IU penicillin G sodium, respectively). Arterial blood was sampled for evaluation of maternal and fetal condition and catheters were flushed with heparinized saline to maintain patency. Animals were 130±1 days GA on the first day of experimental study. Animal care followed the guidelines of the Canadian Council on Animal Care and was approved by the University of Western Ontario Council on Animal Care.

### 2 Experimental procedure

All animals were studied over a ∼6 hour period. After a 1–2 hour baseline control period, mild, moderate and severe series of repetitive UCOs were performed. UCOs were induced in all series by graduated inflation of the occluder cuff with a saline solution. Preliminary studies were performed to determine the amount of volume necessary to achieve mild, moderate, and severe variable FHR decelerations. During the first hour following baseline, mild variable decelerations were performed with a partial UCO for 1 minute duration every 2.5 minutes, with the goal of decreasing FHR by ∼30 beats per minute (bpm), corresponding to an ∼50% reduction in umbilical blood flow [11], [18]–[20]. During the second hour, moderate variable decelerations were performed with an increased partial UCO for 1 minute duration every 2.5 minutes with the goal of decreasing FHR by ∼60 bpm, corresponding to an ∼75% reduction in umbilical blood flow [11], [18]–[20]. Following the moderate variable decelerations, animals underwent severe variable decelerations with complete UCO for 1 minute duration every 2.5 minutes for up to two hours or until the targeted fetal arterial pH of less than 7.00 was detected, at which point the repetitive UCOs were terminated. All animals were allowed to recover following the last UCO for 48 hours prior to necropsy.

Maternal venous blood samples were drawn at baseline and at completion of the UCO protocol. Fetal arterial blood samples were drawn at baseline, at the end of the first UCO of each series (mild, moderate, severe), and at 20 minute intervals (between UCOs) throughout each of the series, as well as at 1, 24 and 48 hours of recovery. For each UCO series blood gas sample, 0.7 ml of fetal blood was withdrawn, while 4 ml of fetal blood was withdrawn at baseline, at pH nadir less than 7.00, and at 1 hour and 48 hours of recovery. The amounts of blood withdrawn were documented for each fetus and replaced with an equivalent volume of maternal blood at the end of day 1. All blood samples were analyzed for blood gas values, pH, glucose, and lactate with an ABL-725 blood gas analyzer (Radiometer Medical, Copenhagen, Denmark) corrected to 39.0°C.

After the 48 hours recovery blood sample, the ewe and the fetus were killed by an overdose of barbiturate (30 mg sodium pentobarbital IV, MTC Pharmaceuticals, Cambridge, Canada). A post mortem was carried out during which fetal gender and weight were determined and the location and function of the umbilical occluder were confirmed.

Normoxic UCO (N/UCO) group animals were defined as those with fetal arterial O2 saturation as measured on post-op days 1, 2, 3, and 4, and on the experimental day during the control period, that on average was>55% (n = 9). Hypoxic UCO (H/UCO) group animals conversely, were those whose O_2_ saturation for these time points on average was <55% (n = 5). An additional group of normoxic animals received E. coli LPS 2 mg bolus infusion into the amniotic cavity via the amniotic catheter starting one hour prior to initiating the UCOs (LPS/UCO, n = 6), *i.e.*, at the beginning of the second hour of the baseline control period, again at the start of the mild UCO series, and continuing hourly thereafter until the UCOs were stopped to simulate a low-grade bacterial infection [21].

### 3 Data acquisition

A computerized data acquisition system was used to record fetal arterial and amniotic pressures, the ECG, ECOG and EEG electrical signals, as previously described (14). All signals were monitored continuously throughout the experiment. Arterial and amniotic pressures were measured using Statham pressure transducers (P23 ID; Gould Inc., Oxnard, CA). Arterial blood pressure (ABP) was determined as the difference between instantaneous values of arterial and amniotic pressures. A PowerLab system was used for data acquisition and analysis (Chart 5 For Windows, ADInstruments Pty Ltd, Castle Hill, Australia). Pressures, ECG, ECOG and EEG were recorded and digitized at 1000 Hz for further study. For ECG, a 60 Hz notch filter was applied, while for EEG, a band pass 0.3–30 Hz filter was used. FHR was triggered and calculated online from arterial pressure systolic peaks. Prior to analysis, ECOG and EEG were sampled down to 100 Hz as is standard practice with fetal ECOG and EEG analyses and to reduce computational requirements for subsequent analysis steps. The focus of this manuscript is on the EEG data in relation to FHR to develop an online algorithm deployable on the bedside. ECOG and ECG data are reported separately.

## Methods

### 1 Detection of abnormal patterns in EEG and FHR

#### 1.1 Long Term and Short Term Averages

FHR is stable except for obvious episodes during which FHR dropped significantly. In the mean time, the EEG is showing cyclic activity that may or may not be regular. From a data analysis point of view, EEG might be considered as more noisy than FHR in the sense that detailed information might not necessarily be very helpful to reach a final decision whether EEG and FHR are synchronized, the criterion indicative of an incipient fetal acidemia. This created a significant difficulty for the traditional statistical analysis. In order to reduce the influence of noise from EEG and FHR, one needs to exact significant and characteristic features from the two time series first.

In order to reconcile the fact that patterns in EEG and FHR are manifested in two different domains, we first need to construct a new statistical measure and try to bring two different data time series into the same and comparable platform. Therefore, we first need to construct a new measure in order to detect any status change in EEG. The new measure should be robust and the influence of the noise level should be minimized. A similar measure should also be applied to FHR to ensure that such a transformation would not introduce any distortions into the relationship between the two time series.

In order to introduce a counting measure or indicator function that is widely used in high frequency day trading, we now define the long term and short averages.

Given a time series X(t), we now define the following quantities
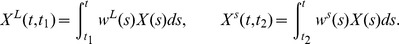
where *t_2_ <t_1_*, and *w^L^ and w^S^* are non-negative weight functions.

**Figure 1:**
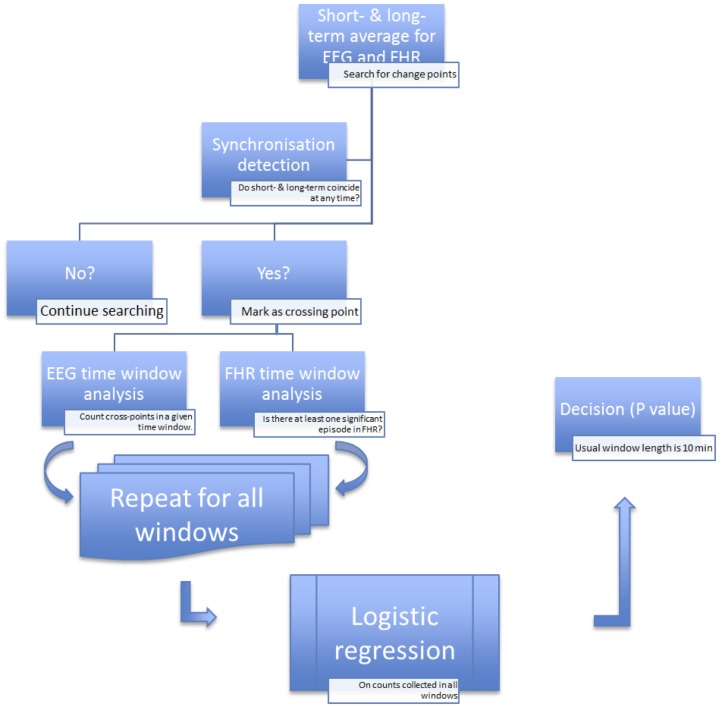
The algorithm to detect EEG-FHR synchronisation.

The long term moving average represents normal and regular cycles in healthy fetuses. The long term moving average at any given time point is defined to be a weighted average of the past by tracing back for a long period of time, 10 min in the current application. The average is indicative of the general trend by minimizing the impacts of short-term bursts or drops in the time series. Since the impact of the oscillation is a relatively local pattern, we need to summarize local information. This is achieved by calculating a short-term weighted moving average in a local manner. Therefore, the two moving averages both possess the ability to smooth out the original time series by noise reduction. However, they represent global or local patterns (Fig. 1).

Our objective is to employ a meaningful measure to reflect or describe the abnormal patterns in the absence of exact knowledge of the nature of these observed patterns.

To define something that is abnormal, we must first know what is normal. The long-term average then serves as the baseline for comparison or yardstick for finding abnormal patterns. Therefore, we need to compare the long-term trend with the short-term tendency in an effort to find those patterns.

#### 1.2 Significant crossing points

We are now ready to define a new measure that can filter out noisy data by only looking at the so-called change-point in high frequency data in a very volatile market. To achieve this, a change-point is detected by looking at locations of the time scales such that the long-term weighted average coincides with the short-term weighted average [22] [23]. This technique has been widely used and proven to be very successful in high frequency trading. The change-points are locations in which a trend reverses its direction. For example, it can be a point where an upward trend changes to the downward trend and vice versa.

We have decided not to use the term “change-point” since EEG does not possess significant upward or downward trend due to its high frequency nature. We thus use the term “crossing points” where an abnormal pattern is detected. Since the underlying mechanism is unknown, we make no attempt to classify the exact nature of those crossing points. To be more specific, an observed abnormal pattern could result from a genuine fetal stress, or some other unknown and undetectable biological reasons.

Therefore the new measure records the number of abnormal patterns by counting the number of crossing points. This can be achieved in two steps. First, we define a new random categorical variable that indicates whether the long-term and short-term trends coincide at each time point.

Namely, we define 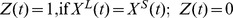, otherwise.

Next, we define the number of crossing points as 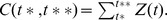


This records the number of times that the long-term trend and the short-term trends equal each other.

Since the entire underlying biological mechanism is not completely understood and could undergo systematic changes, these measures need to be calculated on a continuous basis in order to accumulate enough information or sample size for a subsequent statistical analysis.

### 2 Detection of synchronization

EEG presents a far more significant number of crossing points than FHR. This is because FHR pattern is forced by the UCOs, which, in turn, are induced every 2.5 minutes, while EEG reflects brain's response to the insult. Although FHR might influence EEG, we are making no attempt to establish a causal relationship between both signals. Our main goal is to detect whether the two measures are synchronized at any time point.

We employ a logistic regression model for the detection of synchronization. We set up the response variable as whether or not there is at least one significant crossing point for FHR for any given time window. The length of the time window where EEG and FHR are tested for synchronization is set so that the FHR patterns do not occur more than once in a given time window. The explanatory variable is then set to be the number of significant crossing points for EEG in the same given time window.

A logistics regression is performed to determine whether these two crossings are related by assuming 
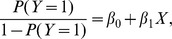
where Y = 1 (presence of anomaly in FHR), with I(.) an indicator function and X the number of significant cross-point in the EEG.

The entire time series of FHR and EEG are divided into 10 minutes non-overlapping time windows with 60,000 time points. The logistic regression is performed on each time window.

The estimation of the coefficients in the logistic regression mode is based on the maximum likelihood method. The hypothesis is that the probability of a significant drop in FHR will not be affected by the knowledge of number of crossing points in the same time window.

In summary, first we divided EEG and FHR time series into 10-minute non-overlapping intervals (Figure 1). Then we identified the crossing points by setting the length of the short-term window and long-term window to be 0.5 s and 2.5 s, respectively. Next, for each interval, we calculated a p-value for testing the null hypothesis that EEG and FHR are not synchronized. We considered the appearance of three consecutive rejections of the null hypothesis as an indicator of synchronization to find the relationship of target synchronized regions to worsening acidemia.

Changes in arterial blood gases and pH versus baseline were analyzed using Friedman repeated measures analysis of variance on ranks followed by multiple comparisons using Dunn's method or repeated measures ANOVA with Holm-Sidak method for multiple comparisons, depending on prior testing for normality. Results are provided as mean±SD and assumed to be significant for p<0.05.

## Results

**Figure 2:**
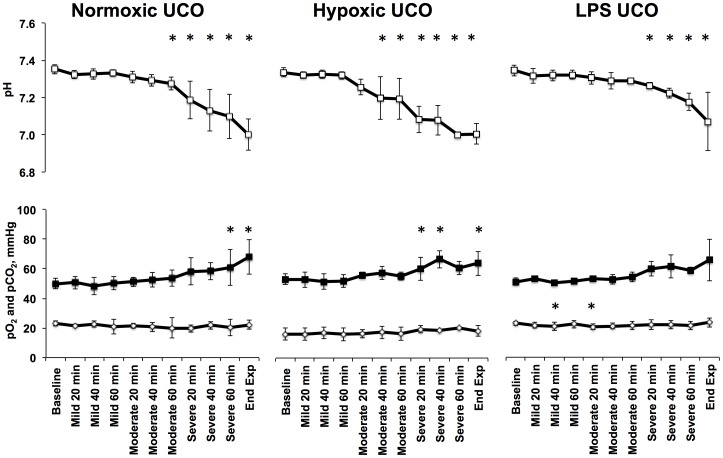
Arterial blood gas values. Mean±SD. UCO, umbilical cord occlusions; values are shown for each 20 min of UCO. * p<0.05 compared to respective baseline values.

In all groups, repetitive UCOs resulted in a pH drop from 7.35±0.03 during baseline to 7.03±0.10 (Fig. 2). The detailed results for the N/UCO group have been presented elsewhere [20]. Averaged across the groups, 77±21 UCOs were conducted per animal with no differences among the groups (Table 1). Table 1 shows the high degree of interindividual variance in the number of UCOs conducted in each ovine fetus. This was due to varying time until pH<7.00 was reached in each animal.

**Table 1:** Number of umbilical cord occlusions (UCOs) in each and all experimental stages.

	Mild UCO	Mod UCO	Severe UCO	Total UCO
**N/UCO**	26±1.5	25±1.2	38±13.7	86±19.5
**H/UCO**	24±0.5	24±0.7	21±12.8	65±18.3
**LPS/UCO**	25±0.0	23±2.3	38±13.3	79±21.7

Mean±SD. N/UCO, normoxic UCO group; H/UCO, hypoxic UCO group; LPS/UCO, normoxic UCO group receiving LPS prior to UCOs.

Fetal arterial blood gas and pH values over the course of the study for the three experimental groups are shown in Figure 2. pO_2_ at baseline for the N/UCO and LPS/UCO groups were within the normal physiologic range, averaging 23.0±1.7 and 23.0±1.4 mmHg, respectively, while that of the H/UCO group was lower, at 16.1±4.0 mmHg (both p<0.05). All three experimental animal groups had normal pCO_2_ and pH values at baseline, which averaged 51.2±3.4 mmHg and 7.35±0.03, respectively. There were no notable significant changes for pO_2_ from its respective baseline values as measured at the end of each of the UCO series for any of the three experimental groups, although a slight drop during mid mild and early moderate UCO series was detected in the LPS/UCO group. pCO_2_ changed little during mild and moderate UCO series, but increased at the end of the severe UCO series for all, but LPS/UCO experimental group (both p<0.05). Fetal arterial pH likewise changed little during mild UCO series, but there was a cumulative decrease in pH with the repetitive UCOs which became significant for the N/UCO and H/UCO groups mid-way through the moderate UCO series, at an average of 7.27±0.04 (both p<0.05); and for all three experimental groups by the end of the moderate UCO series at an average of 7.26±0.07 (all p<0.05). The average time between onset of UCOs and reaching the target nadir pH of 7.00 was shorter in the H/UCO animals at 151±16 minutes compared to the N/UCO animals at 203±14 minutes (p<0.05).

In the LPS/UCO group the target pH of <7.00 was not reached in 3 out of 6 animals due to rupture of the umbilical cord occluder during the severe UCO series, with nadir pHs of 7.25, 7.20, and 7.14. Furthermore, the EEG-FHR synchronization pattern was not evident visually in these three animals. We therefore decided to use these fetuses as our negative controls to test the crossing point method's performance for falsely detecting the EEG-FHR synchronization pattern.

**Figure 3:**
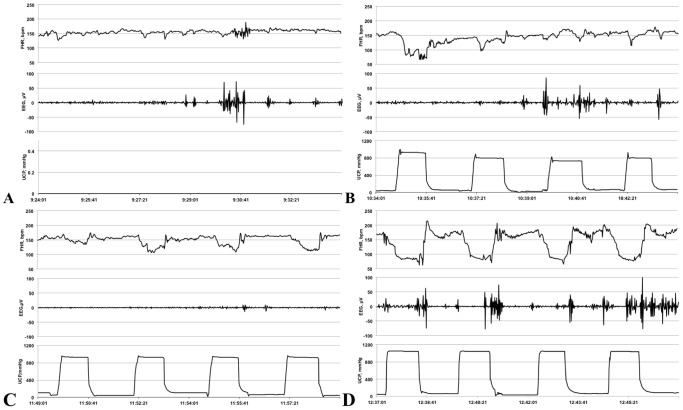
Representative behaviour of the electroencephalogram (EEG) and fetal heart rate (FHR) at baseline and during repetitive umbilical cord occlusions (UCO). 10 minutes of baseline (A), mild (B), moderate (C) and severe (D) UCO series are shown. X axis shows time of the day. The segment during the severe UCO series represents the stage when the adaptive brain shut-down pattern is visible in EEG in phase with FHR decelerations triggered by changes in the umbilical cord occluder pressure (UCP). Note the brief, ∼60 seconds lasting, episodes of EEG suppression during each UCO-induced FHR decelerations and EEG amplitude recovery between the UCOs.

**Figure 4:**
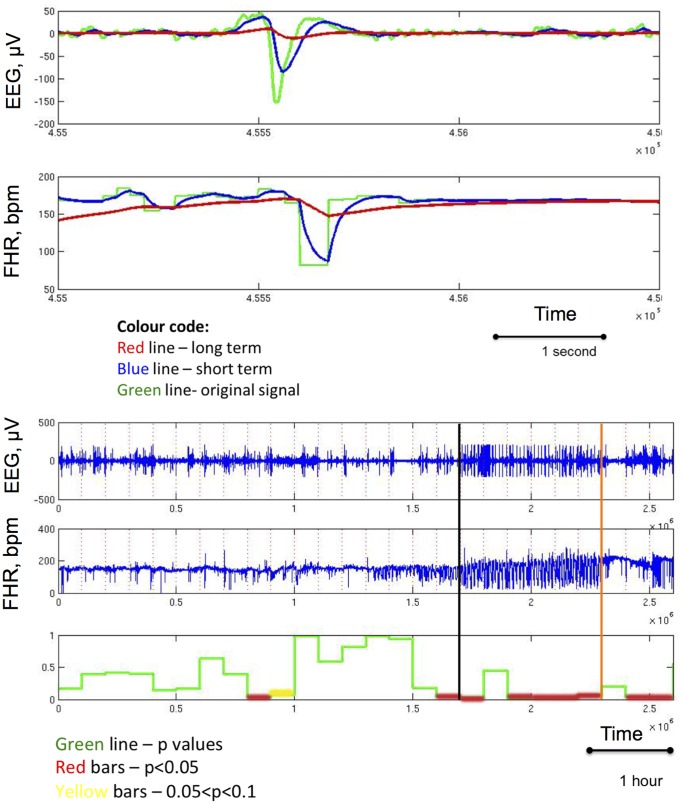
A representative example of crossing point detection. TOP: Single change point and crossing point detection. BOTTOM: Complete experimental recording demonstrating detection of EEG-FHR synchronisation based on the crossing point detection and subsequent validation using logistic regression analysis. Vertical black line denotes onset of EEG-FHR synchronization as per visual expert analysis. Vertical orange bar denotes the drop of pH to less than 7.00. The p-values over time are rendered by red lines where the null hypothesis (no EEG-FHR synchronization) was rejected, *i.e.*, p less than 5% and yellow lines where p value was between 5% and 10%. Note, that three subsequent crossing point detections are required to consider identifying EEG-FHR synchronization. This corresponds to a window length of 10 min (cf. Fig. 1).

Figure 3 shows a representative behaviour of EEG and FHR during the experiment with emergence of EEG-FHR synchronization pattern on average toward the start of the severe UCO series. Figure 4 provides a representative output of the algorithm. In the analysis, we considered the appearance of three consecutive red regions as an indicator of synchronization to find the relationship of target synchronized regions to worsening acidemia. In one case, two consecutive red regions were sufficient (Table 2). The algorithm detected EEG-FHR synchronization 59±31 minutes prior to pH drop, while the “expert” visual detection did so 55±31 minutes prior to pH drop (Table 2).

**Table 2:** Individual algorithm performance.

Animal ID	Algorithm detection (Y/N)	Time prior to pH drop *(based on algorithm)*	Expert detection (Y/N)	Time prior to pH drop *(based on expert)*	Comments
**N/UCO group**					
461060	Y	1:10:00	Y	1:15:06	Noisy
473352	Y	0:33:40	Y	0:37:27	
473378	Y	0:30:00$	Y	0:32:29	Noisy
473361	Y	0:28:20	Y	0:27:59	
5054	Y	0:30:00	Y	0:27:17	
473727	Y	1:55:00	Y	1:52:17	
473377	Y	1:41:40	Y	1:41:51	
473360	N	N/A	Y	0:19:46	Noisy
5060	N	N/A	Y	0:20:41	Noisy
**H/UCO group**					
473351	Y	0:43:20	Y	0:38:29	
473376	Y	1:03:20	Y	1:07:43	
473726	Y	1:11:40	Y	1:17:37	
8003	N	N/A	Y	0:12:20	Too short§
473362	N	N/A	N	N/A	Noisy
**LPS/UCO group**					
4934	N	N/A	Y	0:34	
4935	N	N/A	N	N/A	UCO ruptured♯
5051	N	N/A	N	N/A	UCO ruptured♯
5053	N	N/A	Y	0:37	Noisy
5059	N	N/A	N	N/A	UCO ruptured♯
8002	N	N/A	Y	0:41	

N/UCO, normoxic UCO group; H/UCO, UCO group that was hypoxic prior to UCO start; LPS/UCO, UCO group that received LPS prior to UCO start.

$ Detection based on two subsequent crossing points, as opposed to three elsewhere.

♯ umbilical cord occluder ruptured during the experiments stopping worsening acidemia from developing further; consequently, no adaptive brain shut-down occurred and these foetuses were used as negative controls.

§ EEG-FHR synchronization pattern emerged too shortly prior to pH drop to <7.00 and the algorithm failed to pinpoint even two consecutive positive detections to define synchronization. Clinical benefit of such short time lag prior severe acidemia is also nearly absent, so that this case may represent a limitation of not only the algorithm, but also the clinical utility of the phenomenon itself, even when EEG and FHR are monitored directly visually.

Comparison was then made to the “expert” visual detection of the ECOG-FHR synchronization pattern as previously published [11]. Briefly, as indicated in the Figure 3D, ECOG/EEG-FHR synchronization pattern has been identified as consecutive occurrence of at least three FHR decelerations synchronized in phase with ECOG/EEG amplitude decreases during UCO periods. Although one could study the sensitivity and specificity of these two methodologies, the small sample size renders most statistical tests as not applicable. To answer the question whether the outcome of the algorithm is related or independent of the “expert” visual detection, we set up the null hypothesis that the algorithm prediction and the experts' detection are independent. We applied this analysis to all twenty fetal EEG-FHR recordings. From Table 3, it can be seen that the PPV is 11/16 = 68%, while the negative predictive value is 4/4 = 100%. A detailed break-down for each fetus is provided in Table 2. To test the comparability of the two techniques for detecting fetal acidemia we deployed a commonly used statistics method under the small sample constraint - Fisher's exact test [24]. The null hypothesis is that the algorithm detection is independent of the expert visual detection. The p-value is 0.026 and this indicates that the algorithm is *not in*dependent of the expert visual assessment for detecting fetal acidemia. Therefore, we have sufficient evidence to conclude that the algorithm is consistent with the expert visual assessment for detecting fetal acidemia.

**Table 3:** Comparison of expert detection and prediction by the algorithm.

Algorithm	Detection	No Detection
Expert (Yes)	11	5
Expert (No)	0	4

## Discussion

### 1 Physiological and clinical considerations

Here we introduce a method for online detection of fetal EEG-FHR synchronization pattern during umbilical cord occlusion-induced FHR decelerations as they may be observed during human labour. The EEG amplitude changes during such synchronization pattern consist of brief (∼60 seconds) episodes of EEG suppression during each UCO-induced FHR decelerations and rapid EEG amplitude recovery between the UCOs. With their relatively shorter “on-off” time scale on the order of minutes, such EEG amplitude suppression episodes are different from those reported to occur in fetal sheep in response to UCOs on the order of hours. For example, De Haan *et al.* reported progressive and reversible EEG suppression during worsening acidemia in fetal sheep near-term with a similar UCO pattern paradigm mimicking human labour [25]. Recently, chronic hypoxic ovine fetuses, also subjected to a similar regime of brief repetitive UCOs, were shown to respond with a greater EEG suppression than normoxic fetuses [26]. We do not regard such reversible EEG suppression episodes in response to UCOs as a sign of fetal decompensation. Rather, it is a sign of fetal cerebral adaptive response to repetitive UCOs with worsening acidemia to avoid reaching the point of no return by passing through the lower ischemic flow threshold, which will result in neuronal death [27]. ECOG suppression in the ovine fetus with hypoxia with or without acidemia is a regulated response to offload brain energy needs with decreased oxygen delivery [29]. The response is not related to fetal pH change *per se*, but to a decreased cerebral oxygen delivery and the brain's ability to maintain oxygen consumption. With acute UCOs, ECOG was suppressed by 60–90 seconds of UCO with minimal fetal acidemia [28] [29]. Conversely, with sustained hypoxia over a number of hours and onsetting acidosis, ECOG was slowly suppressed beginning at a pH around 7.15 and did not completely flatten out until fetal pH approached 7.00 [30]. With removal of the hypoxic insult, whether by releasing the cord occluder with UCO or stopping the sustained hypoxic exposure, there can be full recovery of ECOG and presumably brain well-being, as long as the “lower ischemic flow threshold” for cell membrane failure has not been reached.

The observed EEG-FHR synchronization has been suggested to indicate adaptive brain shut-down in response to worsening acidemia and possibly to the cardiovascular decompensation, possibly mediated via endogenous adenosine receptor A1 activation [11] [31] [32]. A timely detection of such adaptive brain shut-down would allow individualized prediction of incipient severe acidemia onset about 60 min prior to its occurrence. Our findings in hypoxic-acidemia EEG and FHR recordings without or with preceding chronic hypoxia or inflammation using chronically catheterized fetal sheep subjected to repetitive UCOs indicate that this method can detect synchronization even with the presence of noisy and non-stationary EEG and FHR signals. To our knowledge, using crossing points from both EEG and FHR to build a statistical model has not been reported, although the crossing point idea has been used in high frequency trading to identify the change of a trend for a single time series [22] [23]. We have now tested and validated an algorithm using crossing point statistical methods for predicting worsening acidosis in a fetal sheep model of human labour compared with visual assessment of EEG-FHR synchronization. In the subset of patients with a non-reassuring FHR tracing during human labour there is need for placement of a FHR scalp electrode and intrauterine pressure catheter to better assess FHR change in relation to contractions. In these patients a double spiral electrode can now be placed to record both FHR and EEG. The results indicate that the proposed method has the potential to improve positive predictive value for detection of fetal acidemia when it is implemented at the bedside. The similarity of this combined FHR/EEG electrode to the presently used single spiral electrode for recording FHR where signal capture has not been an issue with the fetal head usually fixed in the maternal pelvis, should ensure a comparable signal-to-noise ratio and quality of signal for the FHR/EEG electrode.

### 2 Methodical considerations

In our approach, we aimed to make minimal assumptions about the process, as the underlying mechanism is not completely known. This posed several analytical challenges. Commonly used techniques in handling noisy data are the well-known smoothing methods, which use the data in a specified window to generate a smoothed version attempting to filter the noise and capture the true signal. However, smoothing methods are known to be biased at peaks and valleys of a curve and consequently, reduce the sheer magnitude of an episode especially at the points in which a sudden increase or decrease in FHR might occur. Smoothing in EEG creates additional problems since small frequency episodes might be smoothed out and fundamentally alter the underlying characteristics of the frequency domain. Although the EEG and FHR are time series data, the statistical analysis of the relationship of these two data sets is very challenging due to the following reasons. First, the two time series are highly non-stationary and the traditional statistical method for multiple time series requires the assumption of stationarity. This assumption requires the observations to follow the same pattern with the constant mean and variance. The episodes in EEG and FHR would break this assumption since the underlying normal mechanism is altered or overwhelmed by a new one that is our inferential interest. In other words, for FHR, a dramatic drop in the mean due to the occlusion is very evident and expected. The EEG, on the other hand is very noisy and cyclic in nature. The change does not occur for average readings. Instead, the impact of the occlusion can be seen in both the amplitude and more importantly in the frequency domain of the oscillations. To be more specific, we might see different cyclic patterns for the EEG when an oscillation occurs. The traditional methods such as Pearson correlation for independent continuous data are no longer adequate. More advanced methods in time series analysis are also not applicable since the pattern in FHR is in time domain while the patterns in the EEG are in the frequency domain. In summary, the assumptions of traditional regression or longitudinal analysis are violated. Any conclusion derived from applying the classical statistical analysis would be either not sensitive enough to detect the pattern or provide too many false detections. Consequently, these make the test of dependent relationship using the traditional or classical statistical methods very difficult. Thus, many classical statistical methods are not applicable due to violations of the fundamental assumptions for use of these models. The statistical models for change-point detection are mostly parametric in nature and assume that the underlying mechanism can be sufficiently captured by a parametric statistical model. The method based on the change-point detection we present herein handles the non-stationary and highly noisy data allowing detection of EEG-FHR synchronization.

In three of the LPS/UCO animals the target pH of less than 7.00 was not achieved due to rupture of the umbilical cord occluder during the severe UCO series, and no EEG-FHR synchronization was observed. This is consistent with previous observations that a worsening acidemia is necessary to result in adaptive cerebral shut-down manifesting in the EEG-FHR synchronization pattern [11]. Our results show that the method performs very well in the true positive as well as true negative cases as supported by the failed detection of the EEG-FHR pattern in the negative control cases within our cohort. This is important for the eventual bedside application of our approach, as it would ensure that no false positive detection of impending acidemia occurs and hence no false alarm is triggered.

### 3 Limitations of the method

The proposed method is non-parametric in nature and does not rely on a specific model except the logistic regression. However, the expected number of crossing points can be relatively large due to the nature of the function. This could make the decision more difficult as to how many crossing points occurring consecutively should be deemed as significant biologically and represents the limitation of the algorithm as presented here. The limitation is evidenced by the fact that in one instance we required only two, rather than three, crossing points to identify the EEG-FHR synchronization. Generalized or individually specific *a priori* knowledge of the expected number of crossing points or the period of time in the data stream when the occurrence of crossing points ought to be considered biologically significant remain a challenge.

To address this limitation, we could define significant crossing points by selecting relevant crossing points according to a certain criterion. We leave this refinement to future improvement of the proposed algorithm, while the method presented here only verifies whether the two time series are synchronized.

## References

[1] Low JA, Galbraith RS, Muir DW, Killen HL, Pater EA, et al (1984) Factors associated with motor and cognitive deficits in children after intrapartum fetal hypoxia. American Journal of Obstetrics and Gynecology 148: 533–539. 6199975 10.1016/0002-9378(84)90742-7

[2] Goldaber KG, Gilstrap LC 3rd, Leveno KJ, Dax JS, McIntire DD (1991) Pathologic fetal acidemia. Obstetrics and gynecology 78: 1103–1107. 1945216

[3] Winkler CL, Hauth JC, Tucker JM, Owen J, Brumfield CG (1991) Neonatal complications at term as related to the degree of umbilical artery acidemia. American Journal of Obstetrics and Gynecology 164: 637–641. 1992716 10.1016/s0002-9378(11)80038-4

[4] Low JA, Boston RW, Pancham SR (1972) Fetal asphyxia during the intrapartum period in intrauterine growth-retarded infants. Am J Obstet Gynecol 113: 351–357. 4637026 10.1016/0002-9378(72)90683-7

[5] Low JA, Panagiotopoulos C, Derrick EJ (1995) Newborn complications after intrapartum asphyxia with metabolic acidosis in the preterm fetus. Am J Obstet Gynecol 172: 805–810. 7892868 10.1016/0002-9378(95)90003-9

[6] Executive summary: Neonatal encephalopathy and neurologic outcome, second edition. Report of the American College of Obstetricians and Gynecologists' Task Force on Neonatal Encephalopathy. Obstet Gynecol 123: 896–901. 24785633 10.1097/01.AOG.0000445580.65983.d2

[7] Duncombe G, Veldhuizen RA, Gratton RJ, Han VK, Richardson BS (2010) IL-6 and TNFalpha across the umbilical circulation in term pregnancies: relationship with labour events. Early Hum Dev 86: 113–117. 20171025 10.1016/j.earlhumdev.2010.01.027

[8] Chan CJ, Summers KL, Chan NG, Hardy DB, Richardson BS (2013) Cytokines in umbilical cord blood and the impact of labor events in low-risk term pregnancies. Early Hum Dev 89: 1005–1010. 24045131 10.1016/j.earlhumdev.2013.08.017

[9] Liston R, Crane J, Hughes O, Kuling S, MacKinnon C, et al (2002) Fetal health surveillance in labour. J Obstet Gynaecol Can 24: 342–355. 12196870 10.1016/s1701-2163(16)30628-4

[10] Liston R, Sawchuck D, Young D (2007) Fetal health surveillance: antepartum and intrapartum consensus guideline. J Obstet Gynaecol Can 29: S3–56. 17845745

[11] Frasch MG, Keen AE, Gagnon R, Ross MG, Richardson BS (2011) Monitoring fetal electrocortical activity during labour for predicting worsening acidemia: a prospective study in the ovine fetus near term. PLoS ONE 6: e22100. 21789218 10.1371/journal.pone.0022100 PMC3137606

[12] Frasch M, Keen A, Matushewski B, Richardson B (2010) Comparability of electroenkephalogram (EEG) versus electrocorticogram (ECOG) in the ovine fetus near term. Reprod Sci 17: 51A.

[13] Frasch M, Durosier L, Duchatellier C, Richardson B (2012) Fetal sheep electrocorticogram and electroencephalogram changes accompanying variable fetal heart rate decelerations warn early of acidemia. Reprod Sci 19: F–090.

[14] Soothill PW, Nicolaides KH, Campbell S (1987) Prenatal asphyxia, hyperlacticaemia, hypoglycaemia, and erythroblastosis in growth retarded fetuses. Br Med J (Clin Res Ed) 294: 1051–1053. 10.1136/bmj.294.6579.1051 PMC1246217 3107690

[15] Cox WL, Daffos F, Forestier F, Descombey D, Aufrant C, et al (1988) Physiology and management of intrauterine growth retardation: a biologic approach with fetal blood sampling. Am J Obstet Gynecol 159: 36–41. 3394751 10.1016/0002-9378(88)90490-5

[16] Goldenberg RL, Culhane JF, Iams JD, Romero R (2008) Epidemiology and causes of preterm birth. Lancet 371: 75–84. 18177778 10.1016/S0140-6736(08)60074-4 PMC7134569

[17] Becroft DM, Thompson JM, Mitchell EA (2010) Placental chorioamnionitis at term: epidemiology and follow-up in childhood. Pediatr Dev Pathol 13: 282–290. 19888869 10.2350/09-06-0659-OA.1

[18] Frasch MG, Mansano RZ, McPhaul L, Gagnon R, Richardson BS, et al (2009) Measures of acidosis with repetitive umbilical cord occlusions leading to fetal asphyxia in the near-term ovine fetus. Am J Obstet Gynecol 200: 200 e201–207. 19111277 10.1016/j.ajog.2008.10.022

[19] Prout AP, Frasch MG, Veldhuizen RA, Hammond R, Ross MG, et al (2010) Systemic and cerebral inflammatory response to umbilical cord occlusions with worsening acidosis in the ovine fetus. Am J Obstet Gynecol 202: 82 e81–89. 19889382 10.1016/j.ajog.2009.08.020

[20] Ross MG, Jessie M, Amaya K, Matushewski B, Durosier LD, et al (2013) Correlation of arterial fetal base deficit and lactate changes with severity of variable heart rate decelerations in the near-term ovine fetus. Am J Obstet Gynecol 208: 285 e281–286. 23107611 10.1016/j.ajog.2012.10.883

[21] Cheah FC, Jobe AH, Moss TJ, Newnham JP, Kallapur SG (2008) Oxidative stress in fetal lambs exposed to intra-amniotic endotoxin in a chorioamnionitis model. Pediatr Res 63: 274–279. 18091343 10.1203/PDR.0b013e31815f653b

[22] Brock W, Lakonishok J, LeBaron B (1992) Simple Technical Rules and Stochastic Properties of Stock Returns. Journal of Finance 47: 1731–1764.

[23] Brown G (2004) Smoothing, forcasting and prediction of Discrete Time Series: Dover Publications.

[24] Agresti A (2007) An Introduction to Categorical Data Analysis: Wiley-Interscience.

[25] De Haan HH, Gunn AJ, Williams CE, Gluckman PD (1997) Brief repeated umbilical cord occlusions cause sustained cytotoxic cerebral edema and focal infarcts in near-term fetal lambs. Pediatr Res 41: 96–104. 8979296 10.1203/00006450-199701000-00015

[26] Wassink G, Bennet L, Davidson JO, Westgate JA, Gunn AJ (2013) Pre-existing hypoxia is associated with greater EEG suppression and early onset of evolving seizure activity during brief repeated asphyxia in near-term fetal sheep. PLoS One 8: e73895. 23991209 10.1371/journal.pone.0073895 PMC3749175

[27] Astrup J, Symon L, Branston NM, Lassen NA (1977) Cortical evoked potential and extracellular K+ and H+ at critical levels of brain ischemia. Stroke 8: 51–57. 13521 10.1161/01.str.8.1.51

[28] Kaneko M, White S, Homan J, Richardson B (2003) Cerebral blood flow and metabolism in relation to electrocortical activity with severe umbilical cord occlusion in the near-term ovine fetus. Am J Obstet Gynecol 188: 961–972. 12712094 10.1067/mob.2003.219

[29] Kawagoe Y, Green L, White S, Richardson B (1999) Intermittent umbilical cord occlusion in the ovine fetus near term: Effects on behavioral state activity. American Journal of Obstetrics & Gynecology 181: 1520. 10601938 10.1016/s0002-9378(99)70399-6

[30] Matsuda Y, Patrick J, Carmichael L, Fraher L, Richardson B (1994) Recovery of the ovine fetus from sustained hypoxia: effects on endocrine, cardiovascular, and biophysical activity. American Journal of Obstetrics and Gynecology 170: 1433–1441. 8178886 10.1016/s0002-9378(94)70176-8

[31] Hunter CJ, Bennet L, Power GG, Roelfsema V, Blood AB, et al (2003) Key neuroprotective role for endogenous adenosine A1 receptor activation during asphyxia in the fetal sheep. Stroke; a journal of cerebral circulation 34: 2240–2245. 10.1161/01.STR.0000083623.77327.CE 12933964

[32] Frasch MG (2014) Putative role of AMPK in fetal adaptive brain shut-down: linking metabolism and inflammation in the brain. Front Neurol 5: 150. 25157238 10.3389/fneur.2014.00150 PMC4127551

